# Exploring the Therapeutic Potential for Breast Cancer of Phytochemicals and Secondary Metabolites in Marjoram, Thyme, and Persimmon

**DOI:** 10.3390/metabo14120652

**Published:** 2024-11-25

**Authors:** Aubrey Mattingly, Zoe Vickery, Diana Ivankovic, Christopher L. Farrell, Hakon Hakonarson, Katie Nguyen, Luigi Boccuto

**Affiliations:** 1 Healthcare Genetics Laboratory, School of Nursing, Clemson University, Clemson, SC 29634, USA; clf@clemson.edu (C.L.F.);; 2 Center for Cancer Research, Anderson University, Anderson, SC 29621, USA; divanko@clemson.edu; 3 Center for Applied Genomics, Children’s Hospital of Philadelphia, Philadelphia, PA 19104, USA

**Keywords:** plant-based therapeutics, nutraceuticals, phytochemicals, oncology, breast cancer, secondary metabolites, marjoram, thyme, persimmon

## Abstract

**Background/Objectives**: Breast cancer is the most common cause of death in women worldwide and the most commonly diagnosed cancer. Although several therapeutic approaches are widely used against breast cancer, their adverse effects often lead to symptoms severely affecting the quality of life. Alternative methods have been explored to reduce these adverse effects, and nutraceuticals have yielded promising results. This review will discuss mechanisms of action and potential applications against breast cancer of some nutraceuticals, specifically marjoram, thyme, and persimmon leaves. **Methods**: A systematic search was conducted across the public databases of PubMed, PubChem, and Google Scholar, with a specific focus on the plant extracts and phytochemicals of interest, as well as the anticarcinogenic mechanisms. **Results**: Ethnopharmacological and biochemical evidence support the anticarcinogenic role of marjoram, thyme, and persimmon. Numerous phytochemicals contained in these herbs’ extracts, like terpenes and flavonoids, possess remarkable potential to effectively treat breast cancer. **Discussion:** The phytochemicals contained in the reviewed nutraceuticals target the main cellular pathways involved in cell growth and disrupted in carcinogenesis, such as Nf-κB, MAPK/p38, TNF-α/IL-1β, and PI3K/Akt. The mechanisms of action of these compounds can successfully limit the abnormal growth and proliferation of cancerous breast cells. **Conclusions**: The potential use of the phytochemicals discussed in this review, either alone or in combination, may offer a valid alternative to chemotherapy against breast cancer with virtually no adverse effects, and further research on these molecules may lead to the identification of additional chemo-preventative and chemotherapeutic candidates.

## 1. Introduction

Breast cancer is the leading cause of death globally in females and is the most diagnosed cancer [1]. In fact, in the year 2022, the World Health Organization reported that it became the most common cancer in women in 157 countries out of 185 [2]. Current breast cancer treatment options include surgery, radiation, hormone therapy, immunotherapy, and chemotherapy. Although relatively successful, these common therapeutic approaches—especially chemotherapy—create adverse side effects by targeting cells with high basal levels of proliferation [3]. While cancer cells fall into the category of being marked by increased proliferation, so do non-cancerous cells such as skin, hair, and gastrointestinal epithelium [3].

Different approaches for cancer treatment target various aspects of cancer progression. Therefore, the corresponding adverse effects may span a broad spectrum of severity and impact on quality of life: some methods include cell cycle interference, anti-mitotic drugs, traditional chemotherapies, and immunotherapies, among others. Targeting cell-cycle proteins has seen some headway in breast cancer treatment, such as using cyclin-dependent kinase (CDK4 and CDK6) inhibitors, but it is still considered in the early days of application [4]. Other therapeutics include options that work by stabilizing or preventing microtubule assembly like Vinka alkaloids, natural compounds, and those that interfere with mitotic spindle formation, such as taxanes [5], which became an essential part in breast cancer patients in both the advanced and auxiliary settings [6]. Despite having therapeutic efficacy, limitations are reported since tubulin-binding drugs have toxicity in normal tissue, where targeting is not selective to cancer cells [5]. Doxorubicin is a chemotherapy used to treat breast cancer, known colloquially as the “red devil” for the adverse effects it has on patients. To effectively kill breast cancer cells, doxorubicin needs to be highly cytotoxic, which leaves healthy tissue prone to its attack as well. Breast cancer patients who undergo this chemotherapy regimen often experience side effects, which can include induced cardiotoxicity, alopecia, vomiting, oral sores, fatigue, and even death [7]. Other approaches are based on immune responses, creating targets for T-cells so they may attack more efficiently, and this is seen in the use of monoclonal antibodies, cytokines, immune checkpoint blockades, and CAR T-cell therapy. CAR T-cell therapy has been successful in treating blood cancers but has not yet been fully effective up to clinical expectation for solid tumors in breast cancer [8]

Herbal therapeutics and their phytochemical compositions are being studied and gaining attention as potential cancer treatments to reduce these adverse effects and therapeutic limitations [9]. Herbs are the oldest drugs to exist in the world, with medicinal plants representing a pre-existing natural source of remediation that warrants re-visiting. Not only do these natural therapeutics, which will be referred to as nutraceuticals for the remainder of this review, have strong replicated reports that corroborate the phytochemical properties and yield bioactive metabolites that benefit human patients through anticarcinogenic actions and mechanisms [10], but also have proven lower levels of side effects. Complementary and alternative medicine as of 2020 was recorded to be incorporated into patients’ treatment plans and used worldwide, including in 80% of the population in Africa and Asia, even in locations with full access to select modern medical products. This percentage in India is even higher, at 90% [11]. Even still, more research is needed into this nontraditional avenue to provide sufficient evidence and safety backing to increase the usage of herbal therapeutics and alternative medicines in Western populations [11]. Three candidate nutraceuticals, marjoram, thyme, and persimmon leaves, all contain a representative set of phytochemicals that fall into two central classes of molecules (terpenes and phenolic compounds will be discussed). This review highlights and compares them to understand their implications in oncology, specifically for breast cancer. 

Each of these nutraceuticals and their constituents have been investigated in a large range of previous research for exhibiting their anti-cancer properties, and their ethnopharmacological uses will also be briefly reported. The biological effects and mechanisms will be elucidated to pinpoint future molecular targets. As of late, of the 250,000 currently described species in the plant kingdom, only about 10% of medicinal plants have been researched for the treatment of various ailments—and thus, the translational use of nutraceutical therapeutics is still limited [12]. These targets will allow researchers to continue to focus on these promising nutraceuticals as treatment avenues and identify additional chemopreventative and chemotherapeutic candidates with similar composition, containing the same molecular classes, or both.

In this study, we systematically reviewed papers relating to nutraceuticals, specifically marjoram, thyme, and persimmon leaves. Then, we analyzed the ethnopharmacological uses of these nutraceuticals and reviewed the major phytochemical components. We compared and contrasted the classes of compounds within the nutraceuticals and analyzed the implications for breast cancer treatment and metabolomic application. Future directions are considered for translational application to therapeutic delivery for patients.

## 2. Methods

This paper aims to review notable therapeutic potentials, ethnopharmacological uses, and metabolic and mechanistic implications of marjoram, thyme, and persimmon directed toward breast cancer application. As part of this effort, we are attempting to identify phytochemical classes and secondary metabolites associated with these specific nutraceuticals. The Population, Intervention, Comparison, and Outcome (PICO) framework was used to define the focus and scope of the investigation and, subsequently, the literature search strings. This framework was selected for its ability to retrieve well-focused literature based on targeted keywords within databases like PubMed. It is often used to find the answers to evidence-based medicine’s clinical questions [13]. Table 1 presents the PICO characteristics from a broad scope first and then to a narrower focus. These focused strings were used to identify relevant studies based on information contained in the abstracts.

Based on our framework, a systematic search strategy was used for an initial search within PubMed. Independent search strings were used to identify articles regarding marjoram, thyme, persimmon, phenolic compounds, and terpenoids. A complete snapshot of the strategy used (the results for the final strings and at each level of addition of MeSH terms) is available in Table S1 in the Supplementary Material . The search filters applied to the results included English language, full-text available, and published in 2000 or later.

**Figure 1:**
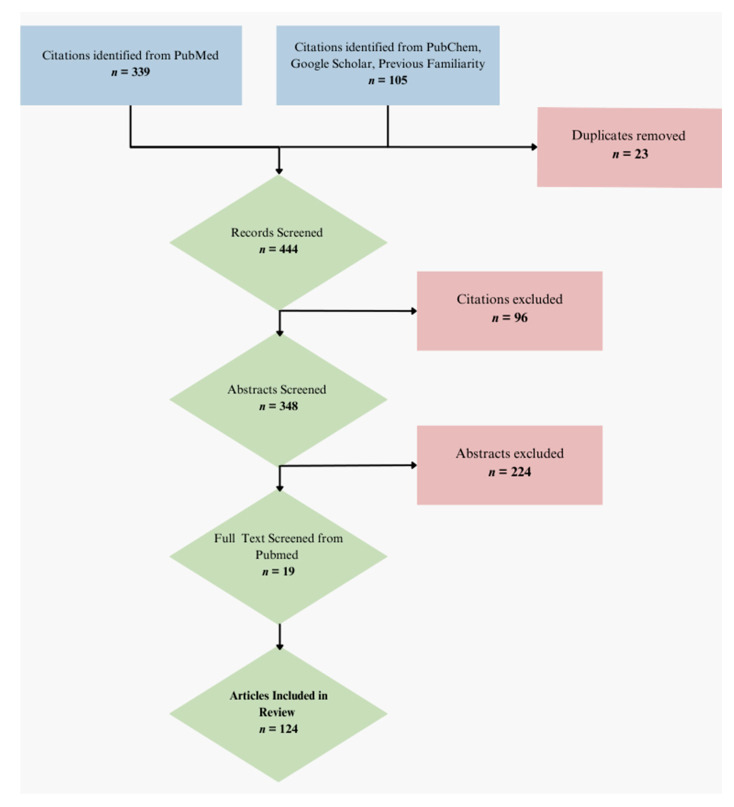
PRISMA-inspired systematic search strategy flow diagram. Citations identified from various databases, duplicates excluded, records screened, abstracts screened, full text screened from search strings, additional papers from PubChem, retrieval results’ references, and previous familiarity. The original pool of records prior to sorting and screening processes are in blue. Removed citations are in red. Finally, in green, it shows articles considered as candidates to include, up to the final set of citations included in the review.

Additional papers were included in the narrative review that authors were previously familiar with, identified within the public databases of PubMed, PubChem, and Google Scholar, associated with the plant extracts and phytochemicals of interest, as well as the anticarcinogenic mechanisms. When investigating specific phytochemicals within the extracts, the “Literature” section of “Consolidated References” in PubChem tabulated components, particularly for notable pathways and pharmacological activities. Figure 1 shows the total number of abstracts screened from the systematic search and the additional papers included in the review through PubChem compilation of recent literature references and familiar sources. The total amount of abstracts screened from the systematic search was 339. Of these screened abstracts, 19 papers were included in our review.

For data extraction, a table was created from each search string into which information from the relevant articles was recorded. For each article, such information included the title, abstract, and descriptive data relevant to the study. The data related to specific categories were summarized using thematic analysis. The recurrent primary themes and sub-categories from independent analyses by both first authors were cross-compared and grouped into clusters and categories, allowing the emergence of a narrative summary presented below.

## 3. Results

Marjoram, thyme, and persimmon are found to contain phytochemicals within both described classes of terpenes (subclasses: monoterpenoids, triterpenoids) and phenolic compounds (subclass: flavonoids) [14,15,16]. Each class of phytochemicals and subclasses underneath are implicated and have been the focus of previous research for exhibiting anticarcinogenic properties, among other related characteristics. Terpenoids are a large class of compounds that occur in most all-natural foods. The subclasses focused on relating to the three nutraceuticals are terpenoids, specifically monoterpenes (thymol, carvacrol, linalool, and limonene) and triterpenes (oleanolic and ursolic acids). Terpenoids are currently being studied as chemo-preventative and chemotherapeutic agents for breast cancer [9]. Phenolic compounds are secondary metabolites commonly found in fruits, vegetables, and spices. They have antioxidant, anti-inflammatory, and anticarcinogenic properties [17]. The subclass of phenolic compounds we are focused on includes flavonoids; those to be mentioned within the nutraceuticals include naringin, hesperetin, isoquercetin, and kaempferol. Flavonoids are polyphenolic compounds with chemo-preventative, anticarcinogenic, and antioxidant properties [18]. Some of these anticarcinogenic properties include the ability to induce cell-cycle arrest, apoptosis, necrosis, and the reduction of multidrug resistance in tumors [19]. Phenolic compounds, within the subclass of flavonoids, are currently being explored for breast cancer treatment [9].

### 3.1. Ethnopharmacological Considerations and Chemical Analysis

- **Thyme** (*Thymus vulgaris* L.): This herb originated in Southern Europe and has been used in traditional medicine for its cardioprotective, gastroprotective, and anti-inflammatory properties [20]. It has also shown evidence of anti-infective, antioxidant, antibacterial, antiviral, and anticarcinogenic properties [21]. The major phytochemicals in thyme include thymol, *p*-cymene, sabinene, carvacrol, borneol, linalool, and limonene (see Table 2). These constituents are all terpenoids and are shown to exhibit antiproliferative and apoptotic effects on cancer cells [22].

**Table 2:** Major phytochemicals contained in thyme.

Phytochemical	Class of Compound	Notable Pathways and Interactions	Pharmacologic and Biologic Activities
Thymol 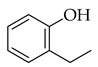	Terpene (monoterpenoid) phenol, isomeric compound to carvacrol	TNF-α, IL-1β, IL-6, NF-κB, TGF-B, PI3K, Akt [23]	antibacterial, antiviral, anticancer, antioxidant, antihypertensive, antifungal, anti-inflammatory, antimalarial [24]
*p*-cymene 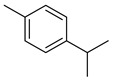	Terpene (monoterpenoid)	TNF-α, IL-1β, IL-6, IL-10, NF-κB, MAPK, ERK1/2, JNK [25]	antioxidant, anti-inflammatory, antiparasitic, antidiabetic, antiviral, antitumor, antibacterial, antifungal, neuroprotective, immunomodulatory, vasorelaxant, analgesic, antinociceptive [26]
Sabinene 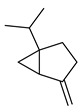	Terpene (monoterpenoid)	TNF-α. IL-1β, OL-6, iNOs, LPS+IFN-y [27]	anti-inflammatory, antifungal, antioxidant [28]
Carvacrol 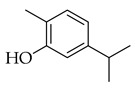	Terpene (monoterpenoid) phenol	IL-1β, IL-6, TNF-α. NF-κB [29]	antibacterial, antiviral, anticancer, antioxidant, antihypertensive, antifungal, anti-inflammatory, antimalarial [24]
Borneol 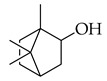	Terpene (monoterpenoid)	p38, MAPK [30] HIF-1α, NF-κB, VEGF, BCL-2 [31]	anti-inflammatory, neuroprotective, anti-apoptotic, and anti-cerebral infarction [30]
Linalool *	Terpene (monoterpenoid)	See Marjoram Components in Table 3 for Details.	
Limonene *	Terpene (monoterpenoid)	See Marjoram Components in Table 3 for Details.	

* These are notable phytochemicals beyond the major components.
- **Marjoram** (*Origanum majorana* L.): This nutraceutical is a medicinal herb originating in the Mediterranean region and traditionally used for its antiallergic, antihypertensive, and antioxidant properties [32]. Additionally, its uses were found in respiratory infections and diabetes; pharmacological analyses showed that this nutraceutical has antioxidant, antifungal, antibacterial, anticarcinogenic, and anti-inflammatory properties [32]. The major phytochemicals in marjoram include terpinene-4-ol, linalool, linalyl acetate, limonene, a-terpineol, naringin, and hesperetin (see Table 3). These phytochemical constituents belong to both classes, terpenoids and phenolic compounds.

**Table 3:** Major phytochemicals contained in marjoram.

Phytochemical	Class of Compound	Notable Pathways and Interactions	Pharmacologic and Biologic Activities
Terpinene-4-ol 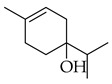	Terpene (monoterpenoid)	KLF4, NF-κB [33], ROCK2 [34], IL-1β, TNF-α, IRAK, IL-17, IL-10 [35]	anticarcinogenic, selective toxicity to cancer cells, when combined with sabinene hydrate, exhibited anticancer effect *in vitro* and *in vivo*, enhancing *survivin* downregulation [36], anti-inflammatory, anti-arthritic, antioxidant [35]
Linalool 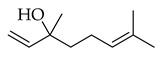	Terpene (monoterpenoid)	VEGF, p-VEGFRII, p-Flk-1, HIF-1α [37] Bax/Bcl-2, caspase-3, caspase-9 [38]	antioxidant, pro-oxidant, anti-angiogenesis, anti-metastasis, anticarcinogenic [37], neuroprotective, alleviation of oxidative stress and apoptosis [38]
Linalyl acetate 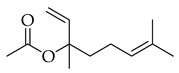	Terpene (monoterpenoid) Acetate ester of linalool	Thymic stromal lymphopoietin and IL-33 [39], Nrf2, NF-κB, p65 [40]	anti-inflammatory, pain modulating [39], antioxidant, anti-apoptotic, protective mechanisms to induction of cancer [40]
Limonene 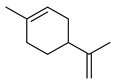	Terpene (monoterpenoid)	TNF-α, IL-1β, COX-2, TLR4, NF-κB, AP-1 [41]	role as a human metabolite, anti-inflammatory, reduced levels of serum urea and creatinine to stop renal decline, reduction of proinflammatory cytokines, modulating oxidative stress [41], anticancer, breast cancer targeting [42]
a-terpineol 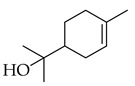	Terpene (monoterpenoid)	KDELC2, Notch, PI3K, mTOR, MAPK [43]	antitumorigenic, antiproliferative, anti-angiogenic, blood–brain barrier penetrable, anti-migration anti-invasion of glioblastoma [42]
Naringin * 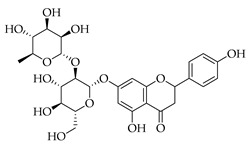	Phenolic (Flavonoid)	PARP-1, ATM, ATR, CHK1, WEE1) [44] GSK3B, NF-κB, COX-2, JAK2, STAT3, Notch1, p38, MAPK, caspase-3 [45]	antioxidant, anticancer, cytotoxic selectivity against cancer cells and not normal cells, free radical scavenging [44,45]
Hesperetin * 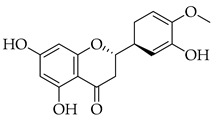	Phenolic (Flavonoid)	IL-1β, TNF-α, NF-κB, caspase-3 [46] PI3K/Akt, tight junction proteins [47]	antioxidant, antineoplastic, anticancer, decreased estrogen receptor (ERα) in breast cancer cells [48], anti-inflammatory, anti-apoptotic, and neuroprotective [46]

* These are notable phytochemicals beyond the major components.
- **Persimmon** (*Diospyros kaki* L.): A fruit-bearing tree whose leaves and other plant components are traditionally used in East Asian countries for a wide application of therapeutics, including for its antidiabetic and antioxidant properties [16]. In traditional Chinese medicine, it is utilized for combating hypertension, hemorrhages, and atherosclerosis [49]. Due to the phytochemicals contained within the leaves, which is the component we are investigating, it is reported to be anticarcinogenic [49,50]. The major phytochemical components in persimmon include oleanolic acid, ursolic acid, pomolic acid, siaresinolic acid, barbinervic acid, astragalin, isoquercetin, and kaempferol (see Table 4). There are more phenolic compounds to note within persimmon than the other selected nutraceuticals—but the highest percentage of the phytochemicals’ mass falls under the terpenes class.

**Table 4:** Major phytochemicals contained in persimmon.

Phytochemical	Class of Compound	Notable Pathways and Interactions	Pharmacologic and Biologic Activities
Oleanolic acid 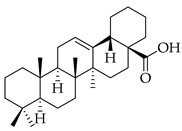	Terpene (triterpenoid)	NF-κB, COX-2, Nrf2 [51], PI3K, Akt, mTOR, p53, MMPs, EGFR [52]	antioxidative, anti-inflammatory, anticancer [51], antifungal, antibacterial, anticarcinogenic, hepatoprotective, gastroprotective, antiviral [53]
Ursolic acid 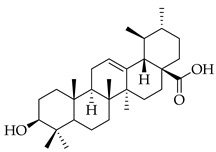	Terpene (triterpenoid)	TNF-α, IL-6, caspase-7, LC3A, LC3B, beclin-1, proapoptotic Bax/Bcl-2, glycolytic pathway, caspase-3, JNK, caspase-9, MMP-2, MMP-9 [54], IL-1β, NF-κB, JNK, MAPK, p65 [55]	antitumor, anticancer, antiproliferative, antiviral, anti-inflammatory, antibacterial, antiallergic, cytotoxic against various cancer cell lines, antiestrogen [54]
Pomolic acid 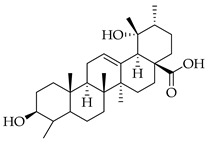	Terpene (triterpenoid)	NF-κB, PI3K, AKT, mTOR, Bcl2, ERK [56], p38-MAPK, HIF-1α, VEGF, p70, S6K [57]	neuroprotective, anti-inflammatory, antioxidant, antiproliferative, free-radical scavenging, anticancer [57]
Siaresinolic acid 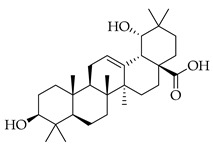	Terpene (triterpenoid)	K(+)ATP channel, TNF-α, IL-1β, CXCL1 [58]	antinociceptive, anti-inflammatory, antiproliferative, antidiabetic [58]
Barbinervic acid 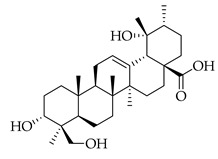	Terpene (triterpenoid)	NF-κB, 5-LO, COX, PDGFR-Rac, JNK, TGF-β1, IL-12, IL-6, VEGF, MMP-9, Na+/K+-ATPase, MAPK, ERK, TNF-α, IL-1β, cGMP (Persimmon) [59]	vasodilator, anti-inflammatory, neuroprotective [60]
Astragalin 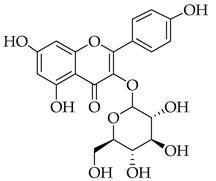	Phenolic (Flavonoid)	HO-1, MAPK, NF-κB, JNK, IL-1β, IL-6, TNF-α, p38, ERK, P13K, Akt, COX-2 [61]	anti-inflammatory, antioxidant, antiallergic, antiviral, anti-neuroinflammatory [61]
Isoquercetin * 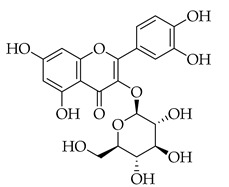	Phenolic (Flavonoid) Glycosidic form of quercetin	Wnt/β-catenin, caspase-3/8/9, p53, Bax/Bcl-2, MAPK [62], Nrf2, NOX4, ROS, NF-κB, p65, p-IκBα, p38, ERK [63]	antitumor, antioxidant, anti-inflammatory, antiproliferative [62], neuroprotective, decrease ROS, anti-apoptosis [63]
Kaempferol * 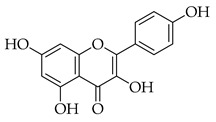	Phenolic (Flavonoid)	ERK1/2, P13K, Akt, mTOR, VEGF, STAT3, p53, NF-κB, TNF-α, IRAK-1/-4, p38, MAPK, ESRRA, HIF-1α, RSK2, COX-2, caspase-3/-7/-9, TYK-2, SOCS-3, MAPILC3, IRE1-CHOP, Bax/Bcl-2, PARP, MMP-9/-2, cathepsin-D/-B, AP1 [64]	anti-inflammatory, anticancer, antioxidant, toxic activities against only cancer cells with restricted toxicity on healthy cells, cardioprotective, neuroprotective, antimicrobial, antidiabetic, enhances apoptosis, inhibits ROS, antiproliferation, cell cycle arrest [64]

* These are notable phytochemicals beyond the major components.

The Supplementary Materials include a complete table (Table S2) of the phytochemicals in marjoram, thyme, and persimmon for further nutraceutical composition elucidation, including chemical properties, structure, and KEGG and PubChem ID.

### 3.2. Terpenoids

Terpenoids are the largest and most diverse group of phytochemicals contained within natural compounds. The amount of terpenoids isolated from various plants is over 40,000 [65]. Terpenoids, also referred to as terpenes, have a variety of chemo-preventative, therapeutic, and pharmacological actions. Since they have diversity in chemical structures and thus functions, bioavailability, and bioactivity, the focus will be on those with fewer isoprenoid units. Terpenoids exist typically as monoterpenoids (structurally having two isoprene units or ten carbons), sesquiterpenoids (three isoprene units, fifteen carbons), diterpenoids (four isoprene units, twenty carbons), or triterpenoids (six isoprene units, thirty carbons) [66]. Nutraceuticals containing terpenoids in the form of mono- and triterpenoids show immense promise for our intended investigation, selected extract contents, and the literature reviewed. Various phytochemicals of both subclasses of terpenoids have been used in many Asian countries, such as in traditional Chinese medicine, to treat headaches, acute fever, and epidemic diseases [67]. It is notable that in addition to monoterpenoids and triterpenoids, diterpenoids and sesquiterpenoids have also shown significant relevant anticarcinogenic activities. Even though these subclasses are not strongly represented in the nutraceuticals for this review, they are worth mentioning to aid in directing future nutraceutical identification and investigation. For example, the widely known Paclitaxel, under the brand name Taxol, is a diterpenoid derived from *Taxus brevifolia* and remains an essential component in breast cancer chemotherapeutics due to its microtubule-stabilizing properties that effectively inhibit cell division [68]. Furthermore, sesquiterpenoids such as β-caryophyllene, commonly found in plants including clove, black pepper, and cannabis, have demonstrated anticancer effects by inducing apoptosis and arresting the cell cycle in cancer cells [69]. Artemisinin, a sesquiterpene lactone from *Artemisia annua*, is also a well-known antineoplastic agent [69]. Terpenoids have been successfully screened for therapeutic efficacy against other ailments such as human immunodeficiency virus and malaria [65], cancer, and, specifically, breast cancer [9].

At the cellular level, terpenoids have been shown to have target-specific capabilities by suppressing proliferation, overgrowth, and excessive erroneous proliferating of cancerous cells being said target [70]. High levels of terpenes contribute to ferric reduction, which is known to be relevant for triple-negative breast cancer tumors and electron scavenging. Iron excess is well understood to contribute to cancer development and is pro-oxidative. Thus, both ferric reduction and electron scavenging mechanisms of terpenes can be important during times of oxidative stress from degenerative disease or cancer progression [71].

#### 3.2.1. Monoterpenes

Monoterpenes are best known as secondary plant metabolites, and if a monoterpene is consumed as a dietary component, it has the potential to prevent tumor formation as well as regress already present malignant tumors [65]. Limonene, a monocyclic terpene found primarily in the isomeric bioactive form of D-limonene, has chemo-preventive properties against rodent mammary cancer during both the initiation and promotion phases of carcinogenesis [65]. D-limonene also proved to have anticancer actions and chemo-preventative actions against early-stage breast cancer, preferentially concentrating in breast tissue after dietary supplementation [72], potentially with induction of apoptosis being through the observed mechanism of cell-cycle arrest at the G2/M phase [73].

Carvacrol is a monoterpene that has been shown to act as an anticarcinogenic agent [22]. It protects against colitis in rats with colon cancer by improving endogenous antioxidants like superoxide dismutase, glutathione, and catalase. Further, it induces cell-cycle arrest at the G2/M phase as well as apoptosis through the downregulation of cyclin B1 and BcL-2 (B-cell leukemia/lymphoma 2 protein) [67]. Both limonene and carvacrol are major components in thyme, while limonene alone is present in marjoram. 

Linalool is another major phytochemical, a monoterpene, found in both marjoram and thyme, with a higher percent of composition in the former nutraceutical (marjoram). According to the literature, multiple studies report that linalool has evidence of anticarcinogenic mechanisms and protective effects on normal cells. Reporting includes its ability to interfere with different intracellular signaling pathways, including the induction of cell-cycle arrest and apoptosis in multiple cancer types [74]. In human prostate cancer cells, cell-cycle arrest at the (sub) G1 phase was demonstrated with an MTT assay [75]. In a separate experiment using the U937 myeloid leukemia cell line, the cell-cycle arrest was also demonstrated to be induced at the G0/G1 phase, where DNA damage accumulated, and subsequently, the tumor suppression mechanisms were activated, inhibiting further proliferation [76]. The common theme of induction of cell-cycle arresting and turning on cell death mechanisms in cancer cells continues with linalool, as it also demonstrated performing this activity in the HeLa cervical cancer cell line. Researchers who applied linalool to the HeLa cell line observed increased expression in genes including *TP53* (encoding the p53 tumor suppressor protein), *CDKN1A* (p21), *CDKN1B* (p27), *CDKN2A* (p16), and *CDKN2C* (p18), all of which facilitate and direct tumor suppression [76]. Knowing that HeLa cells, in their essence, are cervical cancer cells infected with human papillomavirus 18 (HPV-18), linalool must be antiviral since the HPV suppresses *TP53* expression. In terms of selective toxicity to cancerous cells and not healthy cells, pro-apoptotic effects are reported to be limited to cancerous cells with the therapeutic application of linalool [77].

Thymol is the major phytochemical by percent relative concentration in our selected nutraceutical, thyme. It is a monoterpenoid phenol that is also an isomeric compound to carvacrol. Thymol is generally recognized as safe, proven over time, and used for centuries, even dating back to ancient Egypt [78]. This nutraceutical has shown anticancer properties in highly proliferative human cell lines, those mimicking cancer and breast cancer cells in vitro. It has reportedly exhibited potential for being both a chemo-preventative and a chemotherapeutic agent [78]. The mechanistic underpinnings of thymol are described to be antiproliferative, angiogenesis and metastatically inhibitive, pro-apoptotic, and notably cytotoxic to breast cancer cells [79], demonstrated in MCF-7 cells through arresting the cell cycle at the G0/G1 phase [80] and decreasing the viability and proliferative activity of MCF-7 cells [81].

Other research has investigated the complimentary usage of thymol as a combinatorial agent with various chemotherapies to improve the effectiveness of cancer treatment while also minimizing toxicity to normal cells [82]. The activity described is similar to the one reported in linalool and carvacrol. These studies reiterate the potential for the major components within both thyme and marjoram to have minimal toxicity on normal cells with improved effectiveness of cancer treatment, having chemotherapeutic properties specific to breast cancer.

#### 3.2.2. Triterpenes

Triterpenes possess characteristics of notable importance to this investigation as well, including anticarcinogenic and antiproliferative properties with positive therapeutic ramifications [65]. Ursolic acid (UA) and oleanolic acid (OA) are examples of pentacyclic triterpenoids, isomers of one another, found in medicinal herbs from east Asia [65], and are also found in one of our selected nutraceuticals (persimmon) being the highest two phytochemical components by mass. Both UA and OA are reported to overlap with one another in terms of bioactivities [83]. OA shows antitumor and anticarcinogenic properties, with one study suggesting that these characteristics are displayed through the mediation of mitochondrial apoptotic pathways and cell-cycle arresting mechanisms. Additional studies have noted that these characteristics of OA are consistent in cancer growth for different in vitro and in vivo models. In human bladder cancer cells, treatment with OA subdued proliferation and induced apoptosis through the Akt/mTOR/s6K and ERK1/2 pathways, which are critical for cell growth, signaling, and survival [84]. Incubation of OA to adenocarcinomic human alveolar basal epithelial (A549) cells and epithelioid carcinoma (PANC-1) cells exemplified the cell-cycle checkpoint of G0/G1 to be where OA-induced apoptosis occurred [85]. Another specific mechanism of the tumorigenic activity of the triterpenoid OA is the induction of overexpression of the tumor suppressor microRNA miR-122 (miRNA-122 is now understood to regulate cancer cell activities relating to growth, angiogenesis, and migration, facilitating and targeting downstream genetic transcription; expression of miRNA-122 in primary breast cancer cells leading to tumor suppression [86]). A quantification of this in human lung cancer cells in vitro showed that miRNA-122 was induced more than nine-fold following treatment with OA for eight hours [87]. Additionally, researchers showed that OA inhibited the proliferation of hepatocellular carcinoma cells (HepG2) in culture and in vivo with murine models [88]. In this same study with HepG2 cells, it was mentioned that the bioactive properties of OA function through various mechanisms, including upregulating the expression of tumor suppressor protein 53 (p53) and arresting the cell cycle at the G2/M phase [88]. Oleanolic acid methyl ester, a derivative of OA, also exhibited cytotoxic effects on HeLa cells via induction of apoptosis and ROS species production, which was observed to be both in a time- and concentration-dependent manner. In breast cancer with OA application as a therapeutic candidate, proliferation and cell growth were reduced through the prevention of the expression of glycolytic enzymes [89] (which are often upregulated in cancer cells, directing abnormal glucose metabolism and cancer survival in variable environments).

Looking now at ursolic acid, cranberries and blueberries contain this triterpenoid and are functionally similar, related to triterpenoids [90]. The bioactivity of UA was explored in a study that isolated it from the whole cranberry extract and compared it against tumor growth in vitro, finding that two esters of UA inhibited the growth of multiple subtypes, including colon, lung, and cervical cancer cell lines—with a particular selective efficacy exhibited in MCF-7 breast cancer cells. When these researchers isolated UA from the whole blueberry extract, it was screened against leukemia cells, and cell proliferation and DNA synthesis were inhibited. When UA from blueberries was used on human colon cancer and adrenal pheochromocytoma cells at micromolar (µM) concentrations, it continued to display this same action. In another recent research article, UA was demonstrated to act in an antimetastatic manner, increasing the production of ROS, which subsequently led to cell-cycle arrest, mostly at the G0/G1 phase, on two breast cancer cell lines, MCF-7 and MDA-MB-231 [91]. Through these applications and repetition in the literature from research initiatives, we present triterpenoids and the phytochemicals OA, UA and their derivatives at high valuation and potential as anticarcinogenic therapeutic agents against cancer, notably breast cancer.

Several additional pentacyclic triterpenoids are present in the presented nutraceuticals albeit in lower percentages compared to the primary terpenoids discussed; they exhibit significant bioactive properties relevant to cancer treatment and mitigation and are worth mentioning. Pomolic acid, an oleanane-type pentacyclic triterpenoid, has demonstrated potent anticancer activity by modulating key oncogenic pathways, such as NF-κB and PI3K/Akt, which are integral to tumor growth and angiogenesis [56]. Siaresinolic acid, belonging to the ursane skeletal class, is notable for its anti-inflammatory and antinociceptive properties, which could be particularly beneficial in managing cancer-related inflammation; its activity is linked to the modulation of proinflammatory cytokines, including TNF- α and IL-1β [58]. Barbinervic acid, classified under the lupane-type structure, has been reported to possess neuroprotective and anti-inflammatory effects, with evidence supporting its role in inhibiting cancer cell proliferation via the MAPK and NF-κB pathways. Additionally, its vasodilatory properties may reduce cancer-associated complications [92].

### 3.3. Phenolic Compounds

Phenolic compounds are one of the most universal groups of plant metabolites, and more than 8000 compounds have been identified [93]. This class of molecules are secondary metabolites found within most plant tissues [94]. They are not dietary nutrients but instead have bioactive effects, which are of interest. Major classes of bioactive phenolic compounds include phenolic acids, flavonoids, coumarins, quinones, curcuminoids, tannins, stilbenes, lignans, and others [93,95]. Phenolic compounds are reported to have roles in health maintenance and disease prevention through various mechanisms in the body system [96]. At the cellular level, polyphenols prevent protein and DNA damage and inhibit senescent cells to produce senescence-associated secretory phenotype [97].

Recent studies show that polyphenols play an important role in protection against cancer and other diseases known to be related to aging [94]. While the production of oxidative species (hydrogen peroxides and hydroxyl radicals) is part of normal cellular function, the accumulation of these species has also been associated with tumor growth, progression, and aggressiveness [98]. Polyphenols inhibit reactive oxygen species (ROS) production enzymes, such as xanthine oxidase and NADPH oxidase, while also upregulating antioxidants and detoxifying enzymes [93]. Previous research has proven that phenolic compounds have anti-free radical, peroxide decomposer, and oxygen scavenging effects [99], supporting this function against the accumulation of oxidative species and their role in chemoprevention. Polyphenols from plants can promote ncRNAs, which are the main regulators in cancer, making them agents for protecting normal cells whilst killing cancer cells and agents of selective chemotherapeutic effect [93]. Additionally, polyphenols have been shown to inhibit nuclear factor kappa beta (NF-kB), mitogen-activated protein kinase (MAPK), the production of cytokines in inflammatory cells, and toll-like receptors (TLRs), and the downstream proinflammatory gene expression [93]. Phenolic compounds in mushrooms have also been shown to act as anticancer, anti-free radical, peroxide decomposer, and oxygen scavenging agents through the mechanism of activating programmed cell death and inhibiting mediated ROS species reactivity in the NF-kB pathway [99]. Further, previous research gives backing to ROS inhibition, as this mechanism was shown in human colon carcinoma (Caco-2) cells where the antioxidant effect of sweet cherries was demonstrated and directly related to anthocyanin content, a phenolic compound. Sweet cherry extracts had a high phenolic composition and displayed protective effects against oxidative damage caused by species like tert-butyl hydroperoxide [98].

#### Flavonoids

The subclass of phenolic compounds that are contained in all three of the investigated nutraceuticals is flavonoids. As mentioned previously, this set of molecules are plant secondary metabolites that are known for various beneficial properties to human health upon their addition to diet and have been investigated for therapeutic application. DNA methyltransferase (DMNT) activity is directly involved with transcriptional silencing through DNA methylation as well as DNA damage repair, and understandably, aberrations in DNMT activity can increase genomic instability. Flavonoids that have been reported to inhibit this activity include naringin, hesperetin, and quercetin, all phytochemicals found in two of our nutraceuticals (in marjoram for the first two and persimmon for the third), as well as myricetin, curcumin, apigenin, luteolin, garcinol, and hydroxycinnamic acid [93]. Flavonoids have been shown to play a role in chemoprevention as well as chemotherapeutically, acting synergistically with other phytochemicals, including terpenoids and sub-classes, reportedly with blueberries and cranberries—fruits that contain molecules from both classes investigated in this review [90]. Multiple epidemiological studies looking at flavonoid intake and breast cancer incidence have shown an inverse correlation, meaning higher flavonoid intake would equate with lower breast cancer incidence, but there are insufficient and inconsistent data on the epidemiological basis alone [100]. The anti-cancer effect of dietary flavonoids has more robust and sustained evidence in laboratory experimentation. In marjoram and persimmon specifically, naringin, hesperetin, kaempferol, and quercetin are the flavonoids (phenolics) with the most substantial evidence supporting their role as anticarcinogenic nutraceuticals. 

These flavonoids have similar chemical structures and carbon chain backbones but with differences in their attached glycosides. Moreover, bioavailability within the body system differs slightly between phytochemicals. The characteristics of the flavonoids and mechanisms of bioactivity share overlap, highlighting promise for nutraceuticals containing this class of molecules. Naringin is a phytochemical contained within marjoram, a flavonoid with biological and pharmacological activities reportedly including anti-inflammatory, anticancer, anti-apoptotic, anti-atherogenic, antidiabetic, and cholesterol-lowering properties [101]. These characteristics are reported to be through different signal transduction pathways, and investigations have been conducted showing inhibition of tumorigenesis in breast, bladder, and cervical cancer, as well as assistance from naringin paired with current therapies as a combinatorial chemotherapy agent [102]. Regarding biodistribution, the mentioned pharmacological actions have been recorded despite having low oral bioavailability in humans [102]. Researchers applied naringin to triple-negative breast cancer (TNBC) cells, both in vitro and in vivo, and found that the mechanistic bases of the anticancer effects of this phytochemical being, inducing apoptosis and being antiproliferative, were related to cell-cycle arrest at G1 [103]. Further, this investigation demonstrated on MDA-MB-231, MDA-MB468, and BT-459 cell lines that caspase-3 was activated by naringin, p21 (cyclin-dependent kinase inhibitor), and *survivin* (inhibitor of apoptosis) were down-regulated from mediation of the B-catenin pathway, all contributing to decreased viability in the TNBC cell lines. Mice xenografted with human breast cancer cells displayed the same activities associated with a reduction in cancer cell proliferation and tumor size [103]. Hesperetin, an additional flavonoid found in many Asian nutraceuticals—and within our selected nutraceutical marjoram—has been studied after isolation from marjoram and shown in vivo to inhibit multiple types of cancer, including breast, urinary, bladder, and colon cancers [104]. Hesperetin was determined to have strong antiproliferative activities against HeLa cells and rat glioma—which resemble human glioblastoma (C6) cells—at a stronger rate of activity as compared to other phytochemicals extracted from marjoram [104]. The mechanism of inhibiting proliferation was determined in another study, which showed this characteristic in human bladder cancer as well as induction of apoptosis, cellular migration, and invasion by hesperetin via mediation of the PI3K/Akt/FoxO3a pathways and induction of cell-cycle arrest at the G0/G1 phase [105]. An additional flavonoid to note from within the selected nutraceutical of persimmon is kaempferol. This phytochemical is found in a variety of plant sources and traditional medicinal herbs, having marked antitumor, pro-apoptotic, and chemotherapeutic potential across multiple cancer types [106]. In human colorectal cancer cells, it induces apoptosis and displays the action of p53-dependent growth inhibition through various mechanisms [107]. These mechanisms include inhibiting CDK2 and CDK4 [108], the same mechanisms that form the basis of CDK inhibitors used in breast cancer treatment, which are not from natural sources but target cell-cycle proteins. It is also reported that kaempferol acts through cell-cycle arresting at G1 and G2/M phases, resulting from CDK inhibition [108].

Quercetin is one of the most prevalent secondary metabolites in the plant kingdom [97] and is also classified as a flavonoid. It is known to have antioxidative, anti-inflammatory, antiproliferative, and anticarcinogenic properties [94]. A recent study found that quercetin can modulate chromatin modifiers, reducing their activity in a dose-dependent manner and decreasing total DNA methylation [97]. Isoquercetin is a phytochemical contained within the selected nutraceutical, persimmon, and this is a glycosidic form of quercetin. The various glycosidic forms are known to have high solubility and bioavailability [97]. In a study involving sweet cherries, only the phenolic compound quercetin-3,4’-di-O-glycoside could cross the intestinal epithelium without digestion and become bioavailable [109]. Further, quercetin is lipophilic and can reportedly cross the blood–brain barrier with ease, making it a potent curative agent [94]. Clinically, it has been used in nanoparticle and conjugate systems and has improved as an overall anticancer agent. It is reported that quercetin may directly target activation in the Ras–Raf–MEK–ERK pathway. This pathway is aberrantly activated in most types of cancers, as well as the epidermal growth factor receptor family (EGFR). Excessive signaling in EGFR is associated with the development of multiple types of solid tumors [110]. Another research group investigating breast cancer and quercetin found that it induced significant G0/G1 cell-cycle arrest and has been shown to reduce DNA synthesis by 35% in MCF-7 cells when compared to healthy control cells. This same study linked the mechanism of antiproliferative effect to *survivin* gene expression: where the growth of the breast cancer cells was inhibited, the apoptosis was promoted by G0/G1 arrest, and regulation of *survivin* mRNA occurred, which may improve sensitivity to chemotherapy by decreasing the expression in cancerous cells [111].

## 4. Discussion

The evidence collected from the presented literature indicates that the selected nutraceuticals and phytochemicals hold promise for breast cancer treatment. Through describing and characterizing the molecular targets of major phytochemical classes terpenes and phenolic compounds, and sub-classes, monoterpenoids, triterpenoids, and flavonoids contained within marjoram, thyme, and persimmon, therapeutic avenues have emerged that warrant more detail and investigation. It is not disputable that the incidence of cancer is rising at a rapid rate on a global scale, nor is it disputable that there is a need for effective and reimagined therapeutics that reverse, halt, or slow the progression of it [112]. The consideration of metabolic changes characteristic of cancer cells at large and the dynamic metabolic environment of breast tissue is of interest for targets, desired molecular effect, and nutraceutical efficacy following consumption. This research can also help to identify other natural-based therapeutics that would be candidates for displaying these actions and characteristics effectively in the cellular landscape of breast cancer, such as those containing high levels of these same phytochemical classes, subclasses, and specific individual molecules that are explored in this paper. 

**Figure 2:**
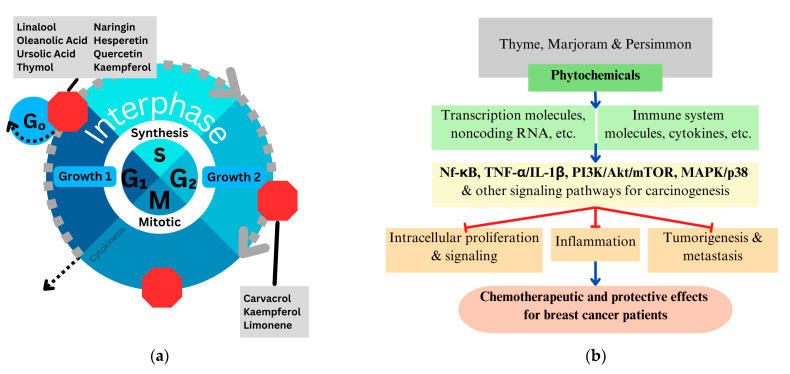
(**a**) Schematic representation of mentioned phytochemicals within monoterpenes, triterpenes, and flavonoids from nutraceuticals with their respective cell-cycle arresting targets. Derived from cited reported experimental models of cancer showing their function at cell-cycle checkpoints (each depicted as a red stop sign), all ultimately resulting in cancer cell death; (**b**) the role in modulation and reduction of nutraceuticals, phytochemicals, in the process of carcinogenesis and on the important signaling pathways within that process, ultimately leading to positive effects for breast cancer patients. Blue arrows represent modulation and promotion, and the red arrows represent reduction.

Pathways and molecules with which all nutraceuticals interact include Nf-κB, MAPK/p38, TNF-α/IL-1β, and PI3K/Akt, as seen in multiple rows in Table 2, Table 3 and Table 4. Targeting these molecules and pathways for breast cancer could offer a multi-faceted approach at various cell cycle checkpoints and modulated pathways, as seen in Figure 2a,b. Nutraceuticals with synergistically active phytochemicals, going after various pathways to stop and prevent cancer progression, represent the multiple facets. 

Nf-κB is a transcription factor with a well-characterized role in inflammatory and immune regulation, cell proliferation, and survival [113]. This pathway is known to be dysregulated in cancer cells of various types and, many times, is constitutively active in breast cancer cells [109]. It is continuously reiterated in molecular targets of the discussed nutraceuticals’ phytochemicals, and it is interconnected with TNF-α and IL-1β, inflammatory cytokines that can activate it. Activation of TNF-α and IL-1β in breast cancer can lead to abnormal genetic transcription, which is beneficial to cancer cells and aids in their resistance to apoptosis, making Nf-κB a critical target in breast cancer therapeutics, particularly in the more aggressive subtypes [114].

The mitogen-activated protein kinase (MAPK) pathway is a complex network of molecules understood as a family of protein kinases involved with signal transduction, cell survival, and cell proliferation. These include extracellular signal-regulated kinases (ERK), c-Jun N-terminal kinase (JNK), and p38, among many others [115]. IL-1β and TNF-α also activate the MAPK pathway, increasing gene expression in those that promote cancer cell growth and inflammation. Notably, other growth factors and cytokines can lead to increased cancer cell survival; interactions of MAPK include Nf-κB and PI3K/Akt signaling axis crosstalk in aberrant breast cells and promote oncogenic processes [116].

The p38 pathway is a signal transducer mediator that is linked to the processes of inflammation, cell cycle, cell death, and tumorigenesis in specific cell types. In mammals, p38 activation is triggered in response to extracellular stimuli such as ultraviolet light, growth factors, and inflammatory cytokines, including those that are further targeted by all nutraceuticals explored in this review, TNF-α and IL-1β. Notably, there is a role of p38 in the translation of the transcripts of TNF-α and IL-1β in inflammatory gene expression. This connection was indicated by steady-state mRNA, which showed no change when protein synthesis was blocked with p38 inhibitors [117]. The p38 pathway additionally plays a role in cell cycle control, regulating proliferation and inactivating CDK inhibitors as tumor suppressors [118]. Crosstalk between the p38 and PI3K/Akt pathways has been studied in the process of cellular differentiation [117].

The PI3K/Akt pathway is a highly complex and interconnected pathway that directs cell proliferation and cellular signaling, responding to locally available hormones, factors, and nutrients. It has been associated with resistance to cytotoxic therapies as well as endocrine and HER2-directed therapies [119]. In breast cancer, inhibition of this pathway is the action of some therapeutics in clinical trials and being repurposed for overgrowth conditions, showing promising data [119]. This pathway is consistently targeted by the nutraceuticals and phytochemicals discussed in this review (see Table 2, Table 3 and Table 4); therefore, these natural compounds may exert similar effects as PI3K/Akt/mTOR inhibitors with additional intracellular targets. This mechanistic hypothesis makes a strong basis for clinical application and the need for further directed therapeutic attention.

The pathways discussed and found to be consistently affected by the reported nutraceuticals in breast cancer-Nf-κB, TNF-α/IL-1β, PI3K/Akt, and MAPK/p38-are interconnected and offer the most relevant molecular targets for therapeutic protocols. The antiproliferative effects on these pathways exerted by the nutraceuticals presented in this review strengthen the therapeutic potential and efficacy of their clinical application. Since multiple pathways are targeted by the nutraceuticals due to the different types of phytochemical constituents, it becomes more likely that escape mechanisms to resistance are overcome because they target different points of these pathways of the intracellular machinery feeding cancer growth. It is worth noting that there are current limitations in clinical validation as most of the work discussed in this review is based upon mainly in silico, in vitro, and in vivo approaches as opposed to well-designed clinical trials. Future clinical trials will be needed to confirm and validate these compelling research findings for the investigated nutraceuticals and their phytochemical constituents potentially as well.

For future applications, there are important factors to consider for broadening the usage of nutraceuticals for breast cancer treatment—especially with thyme, marjoram, and persimmon—and the wide variety of bioactive phytochemicals discussed in this review. First, the type of effective phytochemicals, part of the plant, or whole plant extract, and how it is grown, processed, and extracted. This also ties into ensuring the consistent quality of natural extracts, which is acknowledged as a significant challenge due to inherent variability. Factors such as geographical origin, environmental conditions (e.g., soil, climate, altitude), harvesting times, and post-harvest procession methods all contribute to differences in phytochemical profiles [120]. The concentration of key active compounds such as those in *Thymus vulgaris* L., for a specific example, can vary depending on the environmental conditions in which it is grown. Processing methods can also alter the phytochemical composition such as when drying and extracting, thus affecting the overall bioactivity of the remedy [121,122]. Consequently, quality control is an essential focus in the development and use of phytochemical-based remedies for consistent therapeutic outcomes and standardization [123]. Second, for future consideration, the target population would be breast cancer patients. The target population also includes the subgroups of patients who may change therapeutic options and decisions to employ, such as patient age, stage, and tumor hormone receptor status. Third, the clinical setting for application, such as chemoprevention or chemotherapeutic usage. Also, if it is intended or best utilized in complementary alternative therapy to another anticancer treatment or without any traditional chemotherapy, being substitutive, the last factor to note is dosing. This would include scaling up dosages from animal breast cancer models, which are currently informing clinical breast cancer research and precursors to clinical trials. This also would include determining more about dietary phytochemicals taken orally and the needed amount for effective action and biodistribution to breast tissue and effective deployment. Nanoparticle systems, complexation, different coating materials, and semi-synthetic derivatives are solutions currently being investigated for conjugation to nutraceuticals that widen bioavailability, solubility, and biodistribution [124].

## 5. Conclusions

Natural herbs were the first drugs used by humanity, and ethnopharmacological literature indicates how the knowledge about natural compounds has evolved through centuries in different cultures, eventually merging with modern biochemical notions to highlight the therapeutic potential of numerous nutraceuticals. This review focused particularly on the phytochemicals contained in three nutraceuticals, marjoram, thyme, and persimmon, and their anticarcinogenic effects, with a particular focus on their potential application in treatment protocols against breast cancer. The results emerging from the reviewed literature underline the numerous compounds targeting pathways involved in cell growth and proliferation.

The presence of multiple phytochemicals in the same herbs may allow for synergistic effects on several targets and overcome the drug-resistance mechanisms developed by certain types of cancers. Overall, research on the beneficial effects of herbal extracts offers a different approach to contrast cancer, based on less invasive molecular mechanisms but with combined effects on multiple targets. Such an approach may lead to a better understanding of the role of certain pathways in carcinogenesis, identifying novel targets for treatments, and developing therapeutical protocols with one or more phytochemicals alone or in combination with standard chemotherapeutic drugs to lower the risk of adverse effects.

## Figures and Tables

**Table 1:** PICO characteristics applied to the literature search.

Question Framework Component	Population	Intervention	Comparison	Outcome
Broad Focus	Cancer patients	Alternative herbal treatments for cancer	Currently used cancer treatment strategies	Efficacy, side effects, metabolic targets
Narrowed Focus	Breast cancer patients	Selected nutraceuticals ^1^ with phytochemicals in terpene and phenolic classes	Current chemotherapy regimens for breast cancer	Cancer progression, selective toxicity, pharmacokinetics, biocompatibility, metabolic targets

^1^ Marjoram, thyme, and persimmon.

## Data Availability

The original contributions presented in the study are included in the article/Supplementary Material; further inquiries can be directed to the corresponding author/authors.

## Supplementary Materials

The following supporting information can be downloaded at: https://www.mdpi.com/article/10.3390/metabo14120652/s1, Table S1: Systematic search strategy—tabulated results by string and addition; Table S2: Phytochemical constituents of thyme, marjoram, and persimmon.
