# Supplementary material for: Exploring the Therapeutic Potential for Breast Cancer of Phytochemicals and Secondary Metabolites in Marjoram, Thyme, and Persimmon
Source: Metabolites. 2024 Nov 25;14(12):652. doi: 10.3390/metabo14120652 (PMC11678518; doi:10.3390/metabo14120652)
Supplement: Supplementary file 1 [file metabolites-14-00652-s001.zip › Table S2 Supplementary Material Phytochemical Constituents.pdf]

Phytochemical/ Compound	Molecular Structure	Class of Compound	Molecular Weight (g/mol)	Notable Pathways & Interactions (Reported in Previous Literature)	Pharmacologic and Biologic Activities (Reported in Previous Literature)	KEGG and/or PubChem CID
**Persimmon**						
**Oleanolic acid**	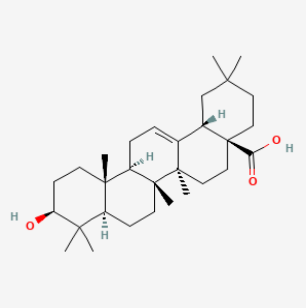	Terpene (triterpenoid)	456.360	**NF-κB**, COX-2, Nrf2 (Baer-Dubowska et al., 2021), PI3K, Akt, mTOR, p53, MMPs, EGFR (Lisiak et al., 2023)	antioxidative, anti-inflammatory, anti-cancer (Baer-Dubowska et al., 2021), antifungal, antibacterial, anti-carcinogenic, hepatoprotective, gastroprotective, antiviral (Castellano et al., 2022)	C17148/10494
**Ursolic acid**	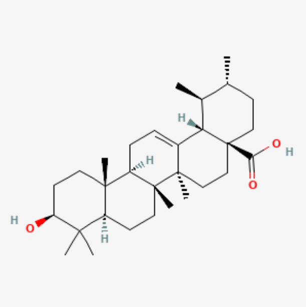	Terpene (triterpenoid)	456.360	TNF-α, IL-6, caspase-7, LC3A, LC3B, beclin-1, proapoptotic Bax/Bcl-2, glycolytic pathway, caspase-3, JNK, caspase-9, MMP-2, MMP-9 (Michalak et al., 2023), Wnt, β-catenin, TGF-β1, STAT3, caspase-3, TCF4 (Zhao et al., 2023) ROCK, PTEN, p53, **NF-κB**, p60, p65, COX-2	antitumor, anti-cancer, anti-proliferative, antiviral, anti-inflammatory, antibacterial, antiallergic, cytotoxic against various cancer cell lines, antiestrogen, (Michalak et al., 2023)	C08988/64945

**Pomolic acid**	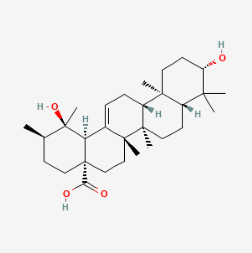	Terpene (triterpenoid)	472.355	**NF-κB**, PI3K, AKT, mTOR, Bcl2, ERK (Martins et al., 2019), p38-MAPK, HIF-1α, VEGF, p70, S6K (Park et al., 2016)	neuroprotective, anti-inflammatory, antioxidant, anti-proliferative, free-radical scavenging, anti-cancer (Park et al., 2016)	C00249/382831
**Siaresinolic acid**	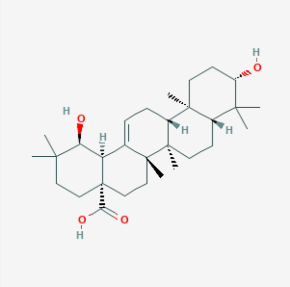	Terpene (triterpenoid)	472.355	K(+)ATP channel, TNF-α, IL-1β, CXCL1 (Marques et al., 2015)	antinociceptive, anti-inflammatory, anti-proliferative, antidiabetic, (Marques et al., 2015)	C17067/123155525
**Barbinervic acid**	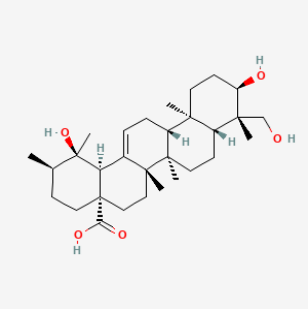	Terpene (triterpenoid)	488.350	**NF-κB**, 5-LO, COX, PDGFR-Rac, JNK, TGF-β1, IL-12, IL-6, VEGF, MMP-9, Na+/K+-ATPase, MAPK, ERK, TNF-α, IL-1β, cGMP (Hossain & Shahidi, 2023)	antioxidant, anti-cancer, antihypertensive, antidiabetic, anti-obesity, anti-tyrosinase, antiallergic, antiglaucoma, (Hossain & Shahidi, 2023)	C03761/194183
**Astragalin**	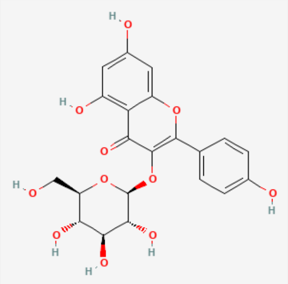	Phenolic (Flavonoid)	448.101	HO-1, MAPK, **NF-κB**, JNK, IL-1β, IL-6, TNF-α, p38, ERK, P13K, Akt, COX-2 (Kim et al., 2022)	anti-inflammatory, antioxidant, antiallergic, antiviral, anti-neuroinflammatory, (Kim et al., 2022)	C12249/5282102
**Persimmon, Notable Phytochemicals Beyond the Major Components**						

**Isoquercitrin**	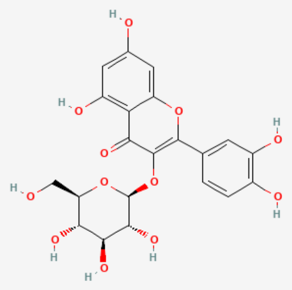	Phenolic (Flavonoid) Glycosidic form of quercetin	464.095	Wnt/β-catenin, caspase-3/8/9, p53, Bax/Bcl-2, MAPK (Wei et al., 2023), Nrf2, NOX4, ROS, **NF-κB**, p65, p-IκBα, p38, ERK (Dai et al., 2018)	antitumor, antioxidant, anti-inflammatory, anti-proliferative (Wei et al., 2023), neuroprotective, decrease ROS, anti-apoptosis, (Dai et al., 2018)	C05623/5280804
**Kaempferol**	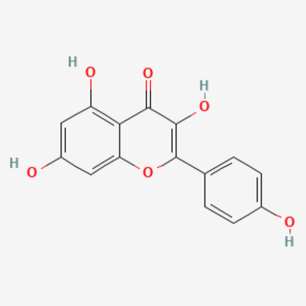	Phenolic (Flavonoid)	286.048	ERK1/2, P13K, Akt, mTOR, VEGF, STAT3, p53, **NF-κB**, TNF-α, IRAK-1/-4, p38, MAPK, ESRRA, HIF-1α, RSK2, COX-2, caspase-3/-7/-9, TYK-2, SOCS-3, MAPILC3, IRE1-CHOP, Bax/Bcl-2, PARP, MMP-9/-2, cathepsin-D/-B, AP1 (Qattan et al., 2022)	anti-inflammatory, anti-cancer, antioxidant, toxic activities against only cancer cells & have restricted toxicity on healthy cells, cardioprotective, neuroprotective, antimicrobial, antidiabetic, enhances apoptosis, inhibits ROS, antiproliferation, cell cycle arrest (Qattan et al., 2022)	na/5280863
**Marjoram**						

**Terpinene-4-ol**	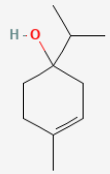	Terpene (monoterpenoid)	154.250	KLF4, **NF-κB**, (He et. al, 2023), ROCK2 (Cao et. al., 2022), IL-1β, TNF-α, IRAK, IL-17, IL-10 (Aslam et. al., 2022)	anti-carcinogenic, selective toxicity to cancer cells, when combined with sabinene hydrate, exhibited anti-cancer effect *in vitro* and *in vivo*, enhancing survivin downregulation (Arafat, 2023) anti-inflammatory, anti-arthritic, antioxidant (Aslam et. al., 2022)	C17013/11230
**Linalool**	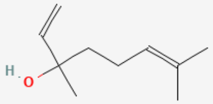	Terpene (monoterpenoid)	154.250	VEGF, p-VEGFRII, p-Flk-1, HIF-1α (Pal et. al., 2022) Bax/Bcl-2, caspase-3, caspase-9 (Hosseini et. al., 2023)	antioxidant, pro-oxidant, anti-angiogenesis, anti-metastasis, anti-carcinogenic (Pal et. al., 2022) neuroprotective, alleviation of oxidative stress and apoptosis (Hosseini et. al., 2023)	C03985/6549
**Linalyl acetate**	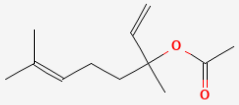	Terpene (monoterpenoid) Acetate ester of linalool	196.290	Thymic stromal lymphopoietin and IL-33 (Lu et. al.,. 2022) Nrf2, **NF-κB**, p65 (Cui et. al., 2021)	anti-inflammatory, pain modulating (Lu. et. al., 2022) antioxidant, anti-apoptotic, protective mechanisms to induction of cancer (Cui et. al., 2021)	na/8294

**Limonene**	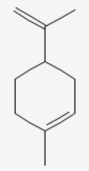	Terpene (monoterpenoid)	136.23	TNF-α, IL-1β, COX-2, TLR4, **NF-κB**, AP-1 (Kathem et. al.,. 2024)	role as a human metabolite, anti-inflammatory, reduced levels of serum urea and creatinine to stop renal decline, reduction of pro-inflammatory cytokines, modulating oxidative stress (Kathem et. al., 2024) anti-cancer, breast cancer targeting (Kaur et. al., 2022)	D00194/22311
**alpha-terpineol**	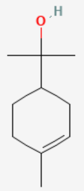	Terpene (monoterpenoid)	154.25	KDELC2, Notch, PI3K, mTOR, MAPK (Jin et. al., 2024)	anti-tumorigenic, anti-proliferative, anti-angiogenic, blood-brain-barrier penetrable, anti-migration anti-invasion of glioblastoma (Jin et. al., 2024)	C16772/17100
**Marjoram, Notable Phytochemicals Beyond the Major Components**						
**Naringin**	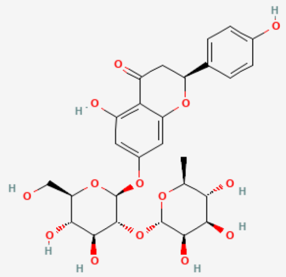	Phenolic (Flavonoid)	580.50	PARP-1, ATM, ATR, CHK1, WEE1) (Pravin et. al.,., 2024) GSK3B, **NF-κB**, COX-2, JAK2, STAT3, Notch1, p38, MAPK, caspase-3 (Rauf et. al., 2022)	antioxidant, anticancer, cytotoxic selectivity against cancer cells and not normal cells, free radical scavenging (Pravin et. al, 2024) (Rauf et. al, 2022)	C09789/442428

**Hesperetin**	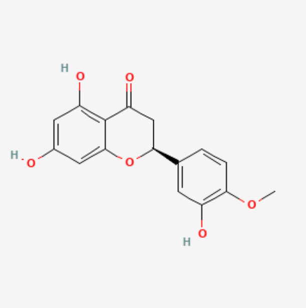	Phenolic (Flavonoid)	302.28	IL-1β, TNF-α, **NF-κB**, caspase-3 (Zhang et. al., 2024) PI3K/Akt, tight junction proteins (Gao et. al., 2024)	antioxidant, antineoplastic, anti-cancer, decreased estrogen receptor (ERα) in breast cancer cells (Vosooghi et. al., 2024) anti-inflammatory, anti-apoptotic, neuroprotective (Zhang et. al., 2024)	C01709/72281
**Thyme**						
**Thymol**	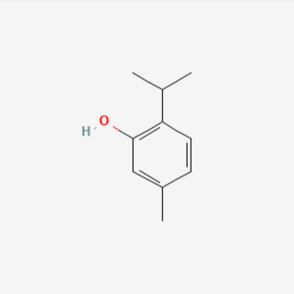	Terpene (monoterpenoid) phenol, Isomeric compound to Carvacrol	150.22	TNF-α, IL-1β, IL-6, **NF-κB**, TGF-B, PI3K, Akt (Hussein et. al., 2024)	antibacterial, antiviral, anti-cancer, antioxidant, anti-hypertensive, antifungal, anti-inflammatory, antimalarial (Peter et. al., 2024)	C09908/6989
**p-cymene**	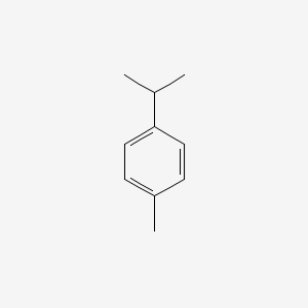	Terpene (monoterpenoid)	134.22	TNF-α, IL-1β, IL-6, IL-10, **NF-κB**, MAPK, ERK1/2, JNK (Zhong et. al., 2013)	antioxidant, anti-inflammatory, antiparasitic, antidiabetic, antiviral, antitumor, antibacterial, antifungal, neuroprotective, immunomodulatory, vasorelaxant, analgesic, antinociceptive (Balahbib et. al., 2021)	C06575/7463

**Sabinene**	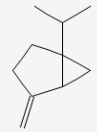	Terpene (monoterpenoid)	136.23	TNF-α. IL-1β, OL-6, iNOs, LPS+IFN-y (Valente et. al., 2013)	anti-inflammatory, antifungal, antioxidant (Cao et. al., 2017)	C16777/18818
**Carvacrol**	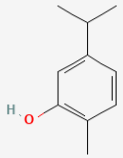	Terpene (monoterpenoid) phenol	150.22	IL-1β, IL-6, TNF-α. **NF-κB** (El Sayed et. al., 2016)	antibacterial, antiviral, anti-cancer, antioxidant, anti-hypertensive, antifungal, anti-inflammatory, antimalarial (Peter et. al., 2024)	C09840/10364
**Borneol**	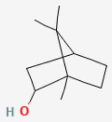	Terpene derivative	154.25	p38, MAPK (Xie et. al., 2023) HIF-1α, **NF-κB**, VEGF, BCL-2 (Chen et. al., 2024)	anti-inflammatory, neuroprotective, anti-apoptotic, anti-cerebral infarction (Xie et. al., 2023)	C01411/64685
**Linalool**	See Marjoram Components for Detail	-	-	-	-	-
**Thyme, Notable Phytochemicals Beyond the Major Components**						
**Limonene**	See Marjoram Components for Detail	-	-	-	-	-
